# In vivo passage of *Salmonella* Typhimurium results in minor mutations in the bacterial genome and increases in vitro invasiveness

**DOI:** 10.1186/s13567-019-0688-1

**Published:** 2019-09-24

**Authors:** Andrea R. McWhorter, Rick Tearle, Talia S. Moyle, Kapil K. Chousalkar

**Affiliations:** 1 School of Animal and Veterinary Sciences, The University of Adelaide, Roseworthy, Australia; 2 Davies Research Centre, School of Animal and Veterinary Sciences, The University of Adelaide, Roseworthy, Australia

## Abstract

Eggs and raw or undercooked egg-containing food items are frequently identified as the bacterial source during epidemiolocal investigation of *Salmonella* outbreaks. Multi-locus variable number of tandem repeats analysis (MLVA) is a widely used *Salmonella* typing method enabling the study of diversity within populations of the same serotype. In vivo passage, however, has been linked with changes in MLVA type and more broadly the *Salmonella* genome. We sought to investigate whether in vivo passage through layer hens had an effect on MLVA type as well as the bacterial genome and whether any mutations affected bacterial virulence. Layer hens were infected with either *Salmonella* Typhimurium DT9 (03-24-11-11-523) as part of a single infection or were co-infected with an equal amount of *Salmonella* Mbandaka. *Salmonella* shedding in both single and co-infected birds was variable over the course of the 16-week experiment. *Salmonella* Typhimurium and *Salmonella* Mbandaka were identified in feces of co-infected birds. *Salmonella* colonies isolated from fecal samples were subtyped using MLVA. A single change in SSTR-6 was observed in *Salmonella* Typhimurium strains isolated from co-infected birds. Isolates of *Salmonella* Typhimurium of both the parent (03-24-11-11-523) and modified (03-24-12-11-523) MLVA type were sequenced and compared with the genome of the parent strain. Sequence analysis revealed that in vivo passaging resulted in minor mutation events. Passaged isolates exhibited significantly higher invasiveness in cultured human intestinal epithelial cells than the parent strain. The microevolution observed in this study suggests that changes in MLVA may arise more commonly and may have clinical significance.

## Introduction

Foodborne gastrointestinal disease is a significant threat to public health and can have substantial economic impacts in terms of cost-of-illness, reduced productivity, and economic losses in both agricultural and food industries. *Salmonella* spp. are among the most common causes of bacterial diarrheal disease in humans [1]. Raw or undercooked eggs or raw egg-containing food items, are frequently linked with infection [2] and are often identified as sources of *Salmonella* during epidemiological traceback investigation of outbreaks [3, 4]. Globally, the two dominant *Salmonella enterica* serovars most commonly associated with egg-related cases of salmonellosis are *Salmonella* Enteritidis (*S.* Enteritidis) and *Salmonella* Typhimurium (*S.* Typhimurium) [2]. In Australia, *S.* Enteritidis is not endemic in commercial poultry farms and the majority of *S.* Enteritidis cases are associated with overseas travel [5]. Furthermore, the incidence of Australian salmonellosis cases linked with the consumption of contaminated eggs has been increasing [4] with definitive phage types of *S.* Typhimurium among the most commonly identified serovar [3].

Phenotypic identification (serotype and phage type) as well as molecular subtyping of isolates are critical for routine surveillance and epidemiological investigation of *Salmonella* outbreaks. One common type of molecular subtyping is multi-locus variable number of tandem repeats analysis (MLVA). MLVA is often used by diagnostic laboratories to characterize the genetic relatedness between bacterial isolates. MLVA subtyping of *S.* Typhimurium is based on the detection of length variations in variable number of tandem repeats (VNTR) loci [6] and involves the PCR amplification of the five most variable VNTR loci (SSTR-3, SSTR-5, SSTR-6, SSTR-9, and SSTR-10) followed by the determination of fragment size through capillary electrophoresis [7]. A MLVA profile is assigned to an isolate based on the number of repeats detected at each of the five loci [8].

MLVA typing is widely used as a molecular epidemiological tool but it has been recently suggested that this method lacks epidemiological sensitivity for certain definitive types of *S.* Typhimurium [9] and that the VNTR loci may be unstable [10–12]. Serial in vitro passaging of strains of *S.* Typhimurium with different MLVA patterns has shown that variations can arise in multiple loci [10–13] and that certain loci are more susceptible to mutation [12]. Instability of MLVA during in vivo passage has also been demonstrated in multiple animal species. Serial passage of a poultry isolate of *S.* Typhimurium DT41 through broiler birds, for example, did not cause a MLVA change but a human isolate exhibited variation at SSTR-10 [10]. In mice, in vivo passage of *S.* Typhimurium DT135a resulted in 4 changes at 3 loci and the rate of mutation in the loci was similar to that of serial passage in vitro [12]. Additionally, serially collected *S.* Typhimurium isolates from human patients also revealed that MLVA type can change with prolonged carriage [13].

Once a layer hen is infected with *S.* Typhimurium it will shed the bacterium intermittently over its lifetime [14]. Layer hens acquire *Salmonella* primarily through the oral-fecal route. Continuous contact with contaminated dust and/or fecal material promotes the likelihood that in vivo passaging of the bacteria will occur in a flock. Limited research has been conducted to characterize the effect serial passaging may have on *Salmonella*. Serial in vivo passage of *S*. Enteritidis through layer hens has been shown to lead to changes in bacterial tropism [15]. It has been hypothesised that the mutation rate of *Salmonella* is higher in vivo than in vitro [16] and as a consequence the emergence of phenotypic variants is likely to arise faster.

The key objectives of this study were to determine if in vivo passage of *S.* Typhimurium through a single “flock” of layer hens can lead to changes in MLVA profile, mutations in the bacterial genome, or bacterial virulence. In these experiments, layer hens were inoculated with either a *S.* Typhimurium DT9 strain singly or with equal proportions of both *S.* Typhimurium DT9 and a *S.* Mbandaka strain. *Salmonella* shedding patterns in feces were monitored over the course of the 16-week experiment. *S.* Typhimurium DT9 isolates were examined for evidence of genomic changes in singly or co-infected layer hens using MLVA typing and whole genome sequencing. Using a human intestinal epithelial cell line, bacterial virulence was investigated for a subset of *S.* Typhimurium DT9 isolates.

## Materials and methods

### Bacterial strains

*Salmonella* Typhimurium DT9 strains are frequently identified during egg related outbreaks of human salmonellosis [3]. Both *S.* Typhimurium DT9 and *S.* Mbandaka are commonly isolated from layer hen farm environments [3, 17]. Thus, isolates of *S*. Typhimurium definitive type (DT) 9 (MLVA 03-24-11-11-523) and *S*. Mbandaka were selected for this study. These strains were originally isolated from the layer farm environment [17]. Strains were stored long term at −80 °C. Prior to use, frozen bacteria were revived on nutrient agar plates overnight at 37 °C.

### Pullet Rearing and layer hen challenge

Fertile commercial brown layer hen eggs were obtained from a hatchery. Eggs were fumigated with formaldehyde prior to incubation to eliminate eggshell surface contamination. Eggs were incubated at 37.7 °C for 21 days. A total of 32 female chicks hatched were raised in an enclosed positive pressure environment to prevent environmental contamination. The facility, all cages, and required equipment were thoroughly cleaned with FoamCleanS and SaniGuard (Chemtall, Australia) and fumigated prior to placement of the birds. Chicks were raised on clean bedding. Feed and water were supplied ad libitum. All feed was treated through fumigation and all water was treated with chlorine tablets (Aquatabs, Ireland). Feed, water, and fecal samples from all birds were collected and processed fortnightly during rearing to ensure that the birds remained free of *Salmonella* prior to infection (method described below).

At 10 weeks of age, birds were divided into three treatment groups: Control (*n* = 4), *S.* Typhimurium DT9 (*n* = 14) and *S.* Typhimurium + *S.* Mbandka (*n* = 14). Each treatment group was contained within separate rooms to prevent cross contamination. Birds were housed individually in cages.

At 14 weeks of age, just prior to the onset of lay, birds in the infection treatment groups were administered 1.0 × 10^9^ colony forming units (CFU) of either *S.* Typhimurium DT9 singly or mixture contained equal amounts of both *S.* Typhimurium DT9 and *S.* Mbandaka (5 × 10^8^ CFU of each serovar) by oral gavage. To generate the inoculum, bacteria were revived from frozen stocks on to nutrient agar. One day prior to the experiment, a single colony was placed into Luria–Bertani (LB) broth and incubated at 37 °C with shaking (110 rpm) for 6 h. Ten microliter of this culture was then sub-cultured into 100 mL LB and incubated overnight with shaking. Bacterial concentrations were determined by measuring the optical density (OD) at 600 nm. Inoculums were diluted to the desired concentration with LB. Control birds received sterile LB. All use of animals was approved by the animal ethics committee of the University of Adelaide (Approval no. S-2014-008) and was compliant with the Australian Code for the Care and Use of Animals for Scientific Purposes.

### *Salmonella* isolation from feces

Fecal samples were collected from each bird at days 1, 3, 6, 9 and 12 and weeks 3, 5, 7, 9, 11, 13 and 15 post-infection (pi). One day prior to each collection, clean plastic was placed below each bird to ensure the collection of fresh feces. Fecal samples were then processed for the presence of *Salmonella* using a double enrichment culture method. Briefly, 10 g of each fecal sample was weighed out and placed in a Whirl–Pak bag (Nasco, USA) containing 90 mL of buffered peptone water (BPW) (Oxoid, Australia) and incubated at 37 °C overnight. 100 µL of this mixture was then placed into 10 mL of Rappaport Vassidalis soya broth (RVS) and incubated at 42 °C overnight. 100 µL of this suspension was then plated on xylose lysine deoxycholate (XLD) (Oxoid, Australia) agar. From each bird at each timepoint, a maximum of 10 *Salmonella* colonies were isolated from XLD plates. These strains were subsequently stored in brain heart infusion (BHI) (Oxoid, Australia) broth with 50% glycerol at −80 °C.

### Isolate identification

DNA was extracted from each isolate using Chelex^®^ (BioRad, Australia). Briefly, 100 µL of an overnight culture was mixed in 900 µL nuclease free water and centrifuged for 2 min at 14 000* g*. The supernatant was removed, and the bacterial pellet was resuspended in 6% Chelex^®^ prepared in TE (10 mM Tris and 1 mM EDTA). Samples were incubated at 56 °C for 20 min, vortexed and then incubated 8 min at 100 °C. Tubes were placed on ice for 5 min and then centrifuged at 14 000* g* for 10 min.

A multiplex PCR described by Akiba et al. was used to distinguish between *S.* Typhimurium and *S.* Mbandaka isolates [18]. Briefly, isolates were confirmed as *Salmonella* through the amplification of an *invA* gene fragment (Forward: 5′-AAACCTAAAACCAGCAAAGG-3′; Reverse: 5′-TGTACCGTGGCATGTCTGAG-3′). Primers designed to the regions *TSR1* (Forward: 5′-ATGCGGGTATGACAAACCCT-3′; Reverse: 5′-TTAGCCCCATTTGGACCTTT-3′), *TSR2* (Forward: 5′-CAGACCAGGTAAGTTTCTGG-3′; Reverse: 5′-CGCATATTTGGTGCAGAAAT-3′) and *TSR 3* (Forward: 5′-TTTACCTCAATGGCGGAACC-3′; Reverse: 5′-CCCAAAAGCTGGGTTAGCAA-3′) were used to differentiate between *S*. Typhimurium DT9 and *S*. Mbandaka isolated from the co-infection group. *TSR3* is not found in *S.* Mbandaka distinguishing those isolates from S. Typhimurium [18]. PCR reactions were conducted in a total volume of 20 µL. Each reaction contained 4 µL 5× MyRed Taq Buffer, 0.5 µM of each forward and reverse primer for *invA*, *TSR1*, *TSR2* and *TSR3*, 0.3 units of MyRed Taq Polymerase (Bioline, Australia), and 2 µL DNA. PCR cycling conditions were as follows: 94 °C for 2 min, 95 °C for 30 s and 60 °C for 30 s repeated for 40 cycles, followed by 72 °C for 5 min.

### Enumeration of bacteria in feces using qPCR

DNA was extracted from all faecal samples collected from birds in both infection treatment groups at all timepoints using QIAamp DNA Stool Mini Kit (Qiagen, Australia) as per manufacturer instructions. Extracted DNA was quantified using a Nanodrop T-1000 (ThermoScientific, Australia) and stored at −20 °C. Further dilution was performed using nuclease free water to achieve a final 5 ng/µL DNA concentration.

The PCR quantification of *Salmonella* was performed using the Quantifast^®^ SYBER^®^ Green QPCR kit (Qiagen, Australia) in a total reaction volume of 10 µL containing 2 µL sample (5 ng/µL), 5 µL of 2× Quantifast SYBER Green Master Mix and 1 µM of reverse and forward primers. The primers to *TSR3* (listed above) were specific for *S*. Typhimurium and were used to quantify this isolate in fecal samples.

In a related study, it was found that the *S.* Mbandaka strain used here was positive for class I integron and contains *dhfrV* (trimethoprim resistance) [19]. The *S.* Typhimurium DT 9 isolate used here tested negative for *dhfrV.* Thus, to quantify *S.* Mbandka in mixed infection samples forward (5′-CTGCAAAAGCGAAAAACGG-3′) and reverse (5′-AGCAATAGTTAATGTTTGAGCTAAAG-3′) primers designed to a region of the *dhfrV* gene were used [20] All reactions were run on a Corbett Rotorgene 6000 (Qiagen, Australia) with the following PCR conditions: 95 °C for 5 min followed by 40 cycles of 95 °C for 10 s and 60 °C for 30 s. All data were analysed using the software version Rotor-gene 1.7.75. To determine the limit of detection and quantification of positive samples, a standard curve was prepared by generating a serial tenfold dilution of faecal samples spiked with various concentrations of *S.* Typhimurium DT9 or *S.* Typhimurium DT9 + *S.* Mbandaka. For fecal samples spiked with *S.* Typhimurium, a cut-off Ct of 33 was used to exclude detection of false positives. A Ct of 33 corresponded to 100 CFU *Salmonella*/g feces. For fecal samples spiked with *S.* Typhimurium + *S.* Mbandaka, a cut-off Ct of 33 was used to exclude detection of false positives. A Ct of 33 corresponded to 1000 CFU *Salmonella*/g feces.

### MLVA typing

MLVA typing was carried out to screen for genomic changes in the MLVA loci. Ten isolates identified by PCR as *S.* Typhimurium DT9 were selected from different hens in each of the infection treatment groups. MLVA typing was performed out by the *Salmonella* Reference Laboratory, Microbiology and Infectious Diseases, South Australia Pathology. The *Salmonella* Reference Laboratory uses the multiplex PCR method developed by Lindstedt et al. targeting the five most variable loci, SSTR-9, SSTR-5, SSTR-6, SSTR-10, and SSTR-3. Briefly, fluorescently labelled primers specifically targeted to the five loci are used to generate PCR fragments [6]. Each MLVA multiplex reaction was run using a touchdown PCR method. MLVA PCR products were run on a gel to check the reaction success prior to sequencing. Products were sequenced by the Australian Genome Research Facility (Adelaide, South Australia).

### Whole genome sequencing (WGS)

DNA was extracted from four *S.* Typhimurium DT9 isolates using the Wizard Genomic DNA Purification Kit (Promega, USA) according to the manufacturer’s instructions. The four isolates included the parent strain, KC109, one isolate from a singly infected bird, and two isolates from separate co-infected birds. One of the isolates from the co-infected birds exhibited a change in MLVA type. A 150-bp paired end library was constructed for each purified bacterial DNA sample and was sequenced using the MiSeq Platform (Illumina) by the Australian Cancer Research Foundation’s Genomic Facility (Adelaide, South Australia). De novo assembly of the reads was performed using Unicycler [21]. Unicycler uses SPAdes [22] for assembly and Pilon [23] for polishing. Bandage [24] was used to visualise assembly graphs and to partition contigs that mapped to the chromosome or plasmids. Genome annotation was carried out using PATRIC [25]. Comparison of ORFs across genomes was carried out using BLAST [26]. Mapping of ORF variants and assessment of their protein consequences was carried out using custom R scripts [27]. The FASTQ files from each sequenced genome have been deposited with the National Center for Biotechnology Information (NCBI) accession number PRJNA559047.

### In vitro bacterial adhesion and invasion assays

The human intestinal epithelial cell line Caco2 (ATCC HTB-37) was used for bacterial adhesion and invasion assays. Cells were be cultured in Dulbecco’s Modified Eagle Media (DMEM) (Hyclone, Australia) containing 4 mM glutamine, 4500 mg/L glucose, 10% (vol/vol) fetal bovine serum (FBS) (Hyclone) and 100 U/mL penicillin and 100 µg/mL streptomycin (LifeTechnologies, Australia) (complete media) at 37 °C with 5% CO_2_. Cells were used between passages 5 to 10.

Changes in bacterial adhesion to Caco2 cells was investigated. This assay was conducted using polarised Caco2 cells that had been previously cultured in growth media, then sub-cultured and placed in 48 well tissue culture trays (NUNC). Caco2 polarization was achieved by maintaining the culture in complete media for 12–14 days. During polarization of the epithelial cell monolayer, tissue culture media was replenished every 48 h.

Two days prior to invasion experiments, bacteria were plated on to XLD agar and incubated at 37 °C overnight. One day prior, stationary phase bacterial suspensions were generated as described above for the in vivo inoculum. Bacteria were quantified by measuring the optical density (OD) at 600 nm. *S.* Typhimurium isolates were added separately to wells of the tissue culture tray to a multiplicity of infection (MOI) of 100 in DMEM containing no supplements. Prior to the addition of bacteria, the cell monolayer was washed three times with DMEM containing no supplements. The *S.* Typhimurium isolates were incubated with the cells at 37 °C for 30 min. Cells were then washed twice with DMEM (with no added supplements). To lift the cells, 150 μL of 0.25% trypsin (LifeTechnologies) was then added to each well and incubated for 15 min at 37 °C. Serial tenfold dilutions were drop plated on XLD agar and incubated at 37 °C overnight. Bacterial colonies were counted, and these numbers were used to calculate the percentage of adhesion for each sample.

Bacterial invasion experiments were conducted using the gentamicin protection assay with polarized Caco2 cells. Polarized Caco2 cells and bacterial suspensions were prepared as described above. Bacteria were added to Caco2 cells at a MOI of 100 and incubated at 37 °C for 2 h; then removed by aspiration. Cells were then washed two times with DMEM containing no supplements. DMEM with 200 µg/mL gentamicin was then added to each well of the tissue culture tray and incubated at 37 °C for 15 min. The media containing gentamicin was removed and the cell monolayers were washed three times with DMEM. Samples were then processed as described above for adhesion.

Both adhesion and invasion experiments were performed three times with four replicates of each isolate.

### Statistical analysis

Statistical analyses were carried out using GraphPad Prism version 8.0. A two-way Analysis of Variance (ANOVA) with a Sidak’s multiple comparisons test was carried out to determine statistical significance of shedding of *S.* Typhimurium DT9 between treatment groups. A D’Agostino and Pearson normality test was performed on adhesion and invasion data. Data were not normally distributed. As a consequence, a Kruskall–Wallis test with a Dunn’s comparison of the means was performed to determine whether the adhesive or invasive capacity of in vivo passaged *S.* Typhimurium DT9 isolates to human intestinal epithelial cells differed statistically. Significance threshold was *p* ≤ 0.05.

## Results

### *Salmonella* collection summary

**Figure 1:**
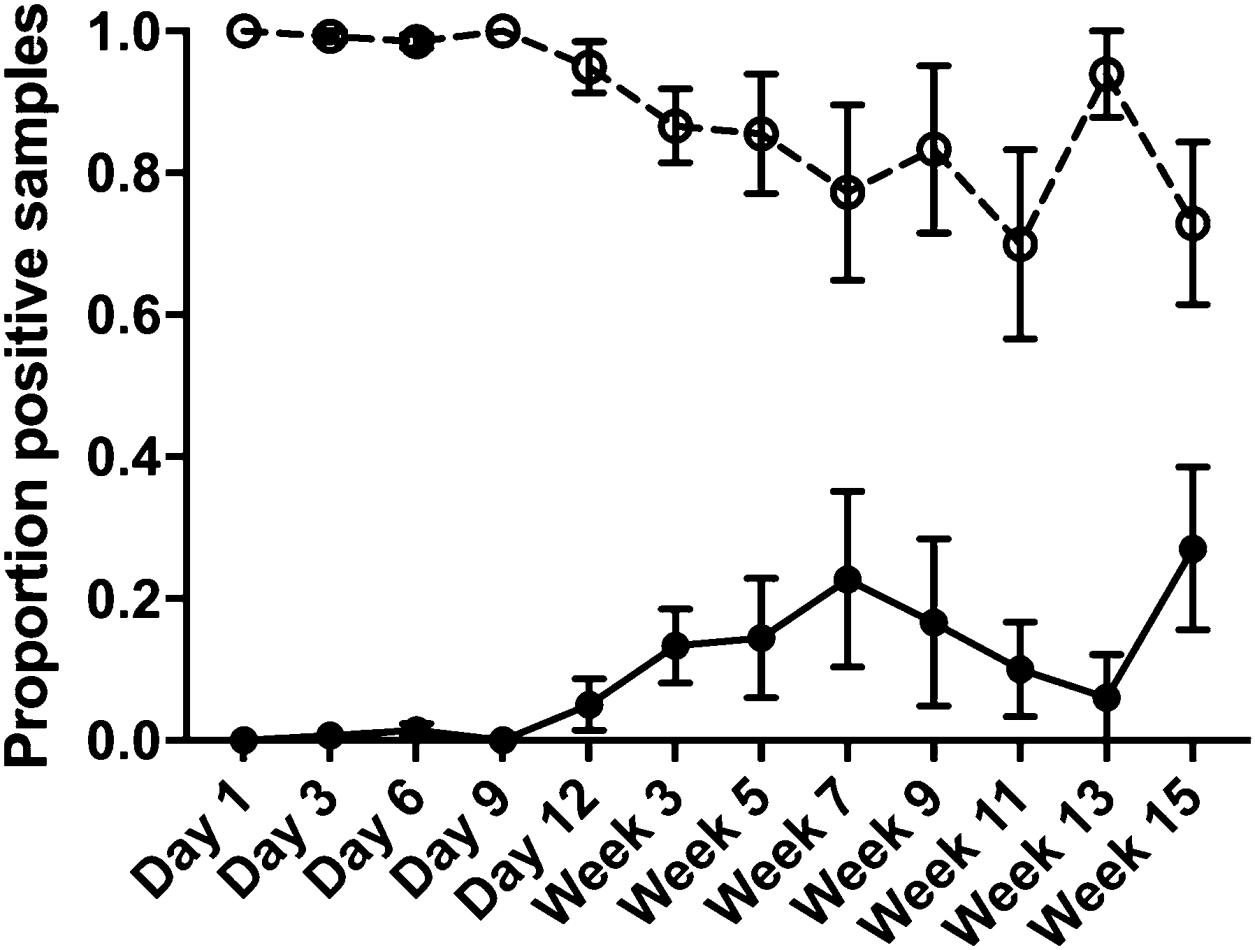
**Proportion of** ***S.*** **Typhimurium and** ***S.*** **Mbandaka isolated from co-infected birds.** Layer hens were infected with an equal proportion of both *S.* Typhimurium DT9 (solid line, filled circle) and *S.* Mbandaka (hashed line, open circle). Fecal samples were collected at each time point. Ten *Salmonella* isolates were collected from each bird and identified using PCR. In co-infected birds, *S.* Mbandaka was isolated at a significantly higher proportion than *S.* Typhimurium (*p* ≤ 0.001).

Over the course of the 16-week infection trial, a total of 1864 individual *Salmonella* colonies were isolated and collected from fecal samples: 925 from birds infected with only *S.* Typhimurium DT9 and 939 from co-infected birds. All isolates collected from birds in the single infection treatment group were confirmed as *S.* Typhimurium. It was expected that co-infected birds would shed both *S.* Typhimurium and *S.* Mbandaka in their feces over the course of the experiment. The proportion of these isolates present post-enrichment was determined using serovar specific PCR. The proportion of each serovar varied over time (Figure 1). During early infection (days 1 through 9 pi), *S.* Mbandaka colonies were found nearly 100% of the time. From day 12 till the end of the experiment, *S.* Typhimurium colonies were also selected but at a much lower proportion (*p* ≤ 0.001) than *S.* Mbandaka (Figure 1).

It should be noted that fecal samples collected from control, uninfected birds were *Salmonella* negative over the course of the entire experiment.

### Quantification of *Salmonella* in fecal samples using a serovar specific qPCR

**Figure 2:**
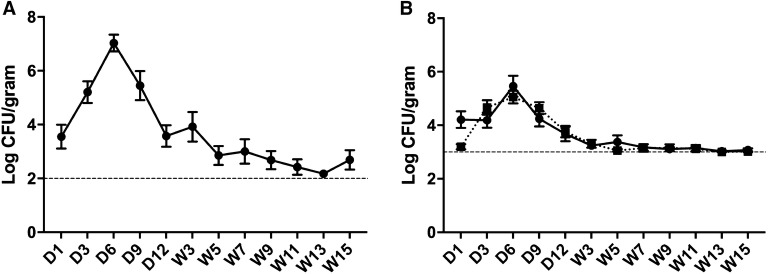
**qPCR assessment of bacteria in feces.** The mean total load of *S.* Typhimurium (solid line, filled circle) in the single infection treatment group **A** as well as both *S.* Typhimurium and *S.* Mbandaka (dotted line, solid square) in the co-infection group **B** were determined using serotype specific qPCR. In both treatment groups, the amount of bacteria shed in feces varied significantly over time (**A**, **B**) (*p* ≤ 0.001). No statistically significant difference was observed in the total amount of *S.* Typhimurium and *S.* Mbandaka shed in co-infected birds. Differences in the limit of detection (hashed line) between the two qPCR reactions prevented a comparison between the shedding of *S.* Typhimurium in singly verses the co-infected birds.

Fecal shedding of *S.* Typhimurium in the single infection treatment group varied over time (Figure 2A) (*p* ≤ 0.001). The mean log_10_ CFU/g feces of *S.* Typhimurium detected by qPCR was highest at day 6 pi (7.03 ± 0.31) and lowest at week 11 pi (2.18 ± 0.11). In co-infected birds, bacterial shedding followed a similar pattern (Figure 2B). At day 6 pi, mean bacterial loads peaked for both *S.* Typhimurium (5.46 ± 0.39 log_10_ CFU/g feces) and *S.* Mbandaka (5.05 ± 0.23 log_10_ CFU/g feces. After week 5 pi, bacterial shedding in the co-infected birds remained just above the limit of detection for the qPCR. Lowest mean log_10_ CFU/g feces for both *S.* Typhimurium (3.03 ± 0.03) and *S.* Mbandaka (3.00 ± 0.01) were observed at week 13. Bacterial shedding also varied over time in co-infected birds (*p* ≤ 0.001) but no statistically significant difference was observed between *S.* Typhimurium and *S.* Mbandaka. Due to the difference in the limit of detection between the two qPCR reactions, a comparison between the shedding of *S.* Typhimurium in the single infection verses the co-infection could not be conducted. Fecal samples collected from control birds were also included in the qPCR assay. All samples were negative for both *dhfrV* and *TSR3*.

### Changes in MLVA pattern

Ten *S.* Typhimurium isolates from each treatment group (single and co-infection) from different time points were submitted for MLVA typing. Colonies isolated at different time points were selected from multiple birds in both the single infection and the co-infection treatment groups. The MLVA type of the parent *S.* Typhimurium DT9 isolate (KC109) was 03-24-11-11-523. All 10 isolates from the single infection group exhibited the same MLVA type as the parent strain. *S.* Typhimurium DT9 isolates from the co-infection collected on days 3, 6 and 9 all retained the parent MLVA type. However, *S.* Typhimurium DT9 isolates from the co-infection group at weeks 9, 11 and 15 pi, possessed an additional six base pair repeat in the STTR-6 locus resulting in an MLVA pattern of 03-24-12-11-523. This change was observed in 7 of the 10 *S.* Typhimurium isolates selected from the co-infection treatment group and was observed in 3 birds.

### Genome sequencing and a summary of genomic features

Four *S.* Typhimurium isolates were selected for whole genome sequencing to determine whether genomic changes arose following in vivo passage in a single or co-infection (Table 1). The parent *S.* Typhimurium strain (KC109) was previously isolated from a layer hen farm environment [17]. Of the passaged *S.* Typhimurium isolates, one (T) was isolated from a singly infected bird at week 9 pi. The other two isolates were collected at day 12 (TM) and week 9 (TMu) pi from two different co-infected birds. The *S.* Typhimurium (T) and one of the co-infection isolates (TM) retained the same MLVA type as KC109 (03-24-11-11-523). The other *S.* Typhimurium isolate from the co-infection (TMu) had the 03-24-12-11-523 MLVA pattern.

**Table 1:** **Isolate summary**

MLVA type	Isolate	Isolation source	Isolation time point
03-24-11-11-523	KC109	Parent strain used in both single and co-infection	N/A
03-24-11-11-523	T	Isolate from a singly infected bird.	Week 9
03-24-11-11-523	TM	Isolate from a co-infected bird	Day 12
03-24-12-11-523	TMu	Isolated from another co-infected bird	Week 9

For the 4 isolates, Illumina 150 nt paired end sequencing was carried out from fragments of average length of 350 base pairs (bp). Genomes for each isolate were assembled de novo. Variants were called and genes annotated. Data for the 3 derived isolates was then compared to the KC109 parent strain. Some basic assembly statistics are listed in Table 2. Overall, the genomes of the four strains were about 4.79 Mb. Considering that de novo assembly generated genomes of 60 contigs > 500 bp for all 4 isolates, the sizes of the chromosomes were remarkably similar, within 0.006% of the parental KC109 (Table 2). Of the 5213 genes found in all isolates, only 10 have altered DNA sequences, and of these only 6 encode altered proteins. All 6 are chromosomal genes. In CDS0910, encoding *rcnA*, a 54-nucleotide (nt) deletion at position 367 removes the amino acid repeat HDHDHDHDHDHDHDHDHD. This deletion occurs in the single infection (T) and the co-infection (TM) isolates but not in the co-infection isolate TMu that is the MLVA type variant. In unknown ORF CDS2064, a G→C substitution at position 334 causes a V→L amino acid change in the TMu isolate only. In CDS4576, encoding a putative surface-exposed virulence protein, an insertion of 66 bp at position 541 causes the addition of 2 copies of an 11amino acid motif that is present in 11 copies in KC109. This insertion occurred in T, TM and TMu. In CDS4779, encoding the Gifsy-1 prophage gene, *recE*, the co-infection isolates TM and TMu exhibited single nucleotide changes at different positions. In TM, a C→T substitution at position 545 causes an A→V amino acid change, while in TMu a T→C substitution at position 374 causes a V→A amino acid change. In the CDS4823, encoding the gene *rha*, a C→T substitution at position 155 causes an A→V amino acid change in TM. In an unspecified prophage protein (CDS4826), an A→C substitution at position 94 causes a S→R amino acid change in TM. The sequence of the STTR-6 loci were located within all of the sequences and with only minor variation were consistent with the MLVA typing results.

**Table 2:** **Sequence summary**

Genome	Chromosome		Plasmid 1		Plasmid 2		Plasmid 3		Plasmid 4		Overall coverage
	Size	Relative coverage	Size	Relative coverage	Size	Relative coverage	Size	Relative coverage	Size	Relative coverage	
KC109	4 794 500	1.04	114 051	0.93	93 562	0.88	4087	6.10	3910	8.45	198
T	4 794 499	1.03	110 331	0.66	93 672	0.76	4087	5.37	3910	5.33	188
TM	4 794 214	0.99	105 380	0.94	93 572	1.09	4087	6.01	3910	4.66	160
TMu	4 794 565	1.00	110 351	0.82	93 672	0.95	4087	5.49	3910	5.75	189

Four plasmids were also identified from each isolate. The plasmid genomes were consistent in size across all 4 isolates. Plasmid 1 had an average genome size of 110 028 base pairs and was almost identical to *Salmonella* plasmid pLST (Table 2). Plasmid 2 had an average size of 93 620 bp and is not similar to any known bacterial plasmid but does show regions of homology to many plasmids. Plasmids 3 and 4 were identical in size in all four isolates at 4087 bp and 3910 bp, respectively. Plasmid 3 is almost identical to several *Escherichia coli* plasmids including pEC542 and Plasmid 4 is identical to pSTY2 (Table 3).

**Table 3:** **Protein changes**

Isolate	Identifier	Gene	Gene product	Change	Position
T, TM, TMu	CDS0910	*rcnA*	Nickel/cobalt efflux transporter	18 AA del	367, 123
TMu	CDS2064		Glutaminyl-tRNA synthetase (EC 6.1.1.18)	V→L	334, 112
T, TM, TMu	CDS4576		Putative surface-exposed virulence protein	22 AA del	541, 176
TM	CDS4779	*recE*	Gifsy-1 prophage	A→V	545, 125
TMu	CDS4779	*recE*	Gifsy-1 prophage	V→A	374, 182
TM	CDS4823		Rha protein	A→V	155, 52
T	CDS4826		Prophage protein	S→R	94, 32

Eight coding sequences found on Plasmid 2 in the parent strain, KC109, were found to be missing in one or more of the passaged isolates. CDS0141, CDS0142, and CDS0143 were found to be absent from TM but not T or TMu. All three passaged isolates (T, TM, and TMu) had deletions of CDS0144, CDS0145, CDS0146, CDS0147, and CDS0148. The absence of these genes was confirmed by PCR using primers designed to the coding sequences of each gene in KC109.

### In vitro bacterial adhesion and invasion

**Figure 3:**
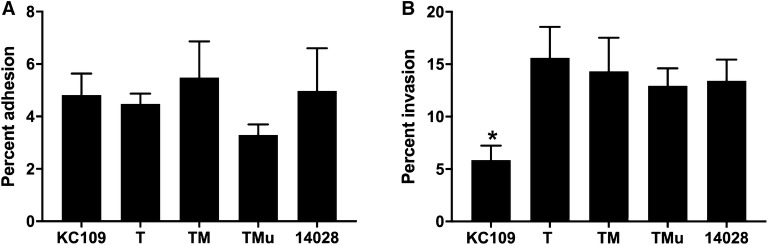
**Invasion and adhesion to human intestinal epithelial cells.** The adhesion (**A**) and invasion (**B**) potential of the parent strain (KC109) and in vivo passaged S. Typhimurium DT9 isolates was investigated using the human intestinal epithelial cell line, Caco2. No significant difference in bacterial adhesion was observed. All passaged isolates, however, were significantly (*p* ≤ 0.05) more invasive than the parent isolate, KC109 (* denotes significance). The control strain, *S.* Typhimurium ATCC 14028 was also significantly more invasive (*p* ≤ 0.05) but did not differ from passaged isolates.

Bacterial adhesion to and invasion into Caco2 cells was investigated to determine whether in vivo passage and/or MLVA change had an effect on aspects of bacterial virulence. Mean percent adhesion ranged between 3.30 ± 0.4 to 5.5 ± 1.4. The isolate with the greatest adhesion was the *S.* Typhimurium DT9 isolate (TM) while the MLVA variant TMu exhibited the lowest adhesion (Figure 3A). No significant difference in adhesion was observed.

The invasive capacity of the isolates was also investigated. The mean percent invasion ranged between 5.8 ± 1.38 and 15.6 ± 2.96. All passaged isolates (T, TM, and TMu) exhibited significantly higher invasion than the parent *S.* Typhimurium isolate, KC109 (*p* < 0.05) (Figure 3B). The ATCC control strain S. Typhimurium 14028, also was significantly more invasive than KC109 (*p* ≤ 0.05) but did not differ significantly from the passaged isolates (Figure 3B).

## Discussion

The experiments presented in this study were designed to investigate the influence in vivo passage through layer hens may have on the genome of a *S.* Typhimurium DT9 isolate. Additionally, we assessed whether the observed changes were associated with changes in bacterial virulence. Over the 16-week infection trial, the total *Salmonella* load was quantified by qPCR in fecal samples of infected birds. Bacterial loads peaked at day 6 post-infection in both single and co-infection treatment groups and, while variable, decreased until the end of the trial. These results are consistent with previous *S.* Typhimurium infection trials in layer hens where shedding peaks early after infection and then decreases over time [14, 28]. In birds co-infected with both *S.* Typhimurium DT9 and *S.* Mbandaka, no significant difference was observed in the total load of each serotype in fecal samples. Due to differences in the limit of detection, the quantification of *S.* Typhimurium from the single infected treatment group could not be directly compared with the loads of *S.* Typhimurium in co-infected birds. It has previously been shown that fecal samples contain PCR inhibitors that may contribute to the reduced efficiency of the reaction [29]. Furthermore, in the presence of PCR inhibitors as well as DNA of other intestinal microbes, competition may arise between reactions in a multiplex PCR, reducing the sensitivity [30].

Both serotypes were isolated from all co-infected birds indicating that there was no impact of co-infection on host colonization. Similar results have been obtained in birds co-infected with *S.* Enteritidis and *S.* Kentucky using different routes of infection [31]. The disparity in the proportion of *S.* Mbandaka and *S.* Typhimurium isolates selected is likely due to colony size variation between the serovars. It has recently been shown that *S.* Mbandaka can have significantly larger colonies than *S.* Typhimurium when plated on to XLD agar [32]. This size variation may have contributed to ease of isolation thereby biasing the selection of *S.* Mbandaka over *S.* Typhimurium.

MLVA typing and whole genome sequencing methods were used to determine whether in vivo passage had an effect on the stability of the *S.* Typhimurium genome of a single clone. Twenty isolates of *S.* Typhimurium DT9 each from either a single (*S.* Typhimurium only) infection or co-infected (*S.* Typhimurium and *S.* Mbandaka) treatment group were submitted for MLVA typing. The 10 isolates submitted from the single infection treatment group all exhibited the same MLVA type as the parent strain. Seven of 10 isolates selected from the co-infection treatment group possessed one additional tandem repeat in the STTR-6 locus. SSTR-6 is known as one of the most unstable VNTR loci [11] so it is unsurprising that this variant was observed. Isolates of the alternate MLVA type were collected at weeks 9, 11 and 15 post-challenge. The 3 parental MLVA isolates were collected at days 6 and 12 post-infection. The 7 isolates with the alternate MLVA pattern originated from three of fourteen birds within the treatment group. We cannot state with certainty whether the mutation in STTR-6 arose during the preparation of the inoculum in vitro or whether it occurred in vivo. Moreover, we cannot determine whether the mutation arose independently in vivo or the mutant strain passed between birds. All birds were housed in individual cages and contact between birds was minimal. The STTR-6 variant isolate, however, may have been spread across cages by handlers or through dust. To our knowledge, there is no existing literature documenting the simultaneous occurrence of identical MLVA changes in individually housed animals.

Layer hen farms often have more than one *Salmonella* serotype and the data presented here suggests that co-infection may contribute to the destabilization of the MLVA. To confirm this, additional in vitro and in vivo experiments would need to be conducted to determine if there were changes in the rate of mutation on the different VNTRs used for MLVA typing of *S.* Typhimurium.

Whole genome sequencing revealed that the isolates were, in general, very similar. Similar results have been found with isolates collected from humans with prolonged carriage of *S.* Typhimurium [13]. In the core genome, mutations in 6 coding sequences resulted in changes in the predicted amino acid sequence. In TMu, the valine to leucine substitution in CDS2064 is likely to have a neutral effect on protein function. Valine and leucine are both aliphatic and hydrophobic amino acids and substitutions with amino acids of the same type are generally favored [33]. Similarly, the amino acid substitutions in CDS4779 (*recE*) observed in TM and TMu are not likely to affect protein functionality. Valine and alanine substitutions are generally regarded as neutral [33]. This is also likely to be true for the A→V amino acid change in CDS4823 in TM. In the TM isolate, a serine to arginine substitution was observed in CDS4826, a putative prophage protein. While both amino acids are polar, this substitution is not likely to be neutral. Arginine has a large positively charged side chain and is generally involved in protein structure [33]. Serines, however, are commonly located in regions involved in protein function [33], so the amino acid substitution in CDS4826 may affect protein functionality, a possibility that requires further investigation.

Two major chromosomal mutations were observed in all three passaged strains. In CDS0910, *rcnA*, a 54-nucleotide deletion was observed in two passaged isolates, T and TM. *RcnA* encodes an efflux pump involved in nickel and cobalt detoxification [34]. The HDHDHDHDHDHDHDHDHD repeat is a nickel and cobalt binding site [35] and deletion of this region in T and TM may alter bacterial sensitivity to these metals. The other major chromosomal mutation was observed in CDS4576 which encodes a putative surface-exposed virulence protein. An additional 2 copies of an 11 amino acid motif were detected in all three passaged isolates, T, TM, and TMu. Because CDS4576 has not been studied, it is not clear what effect this may have on bacterial virulence if any.

Other mutations were observed in Plasmid 2. Eight genes in TM and 5 in T and TMu were found to be absent. Plasmid 2 exhibited some sequence homology with the *Salmonella* plasmid pLST but it is otherwise is unknown. The eight genes were predicted to be involved with antibacterial resistance and plasmid replication but how these mutations may affect the plasmid, or the bacterial isolates is unknown.

Aspects of *Salmonella* tropism [15] and virulence [36] can be affected by in vivo passage. Thus, to determine whether there were changes in aspects of virulence, in vitro bacterial adhesion and invasion were tested using the passaged isolates from this study. All isolates exhibited similar adhesiveness. The passaged isolates, however, all had significantly greater invasive capacity than the parent isolate, KC109. These results are in contrast to another study that did not observe any significant effect of passage on in vitro virulence [37]. No mutation providing an obvious functional reason for the difference observed in invasive capacity was observed. Significant variation in invasion has been shown to occur between isolates of *S.* Typhimurium of different definitive types [38]. Despite having high sequence homology, these different DTs of *S.* Typhimurium also exhibited significant differences in causing disease in mice [38]. Thus, to determine the full effect of the mutations on bacterial virulence, the isolates included in this study require further investigation.

Here we have shown that in vivo passage had limited effect on the genome of *S.* Typhimurium DT9. Both the parental MLVA type, 03-24-11-11-523, and the variant, 03-24-12-11-523, have been isolated during *Salmonella* outbreaks in Australia [39, 40]. Our results are consistent with a previous report demonstrating that the diversity of the *S.* Typhimurium population remains relatively constant over time [41]. The microevolution observed during this study has clinical relevance and should be considered during epidemiological investigation. These results also suggest that changes in MLVA may arise more commonly and from multiple sources. Thus, a broader investigation of changes in MLVA type and genome variation through the food supply chain is required.
